# The three-dimensional culture of L929 and C2C12 cells based on SPI-SA interpenetrating network hydrogel scaffold with excellent mechanical properties

**DOI:** 10.3389/fbioe.2023.1329183

**Published:** 2024-01-10

**Authors:** Chunmin Ma, Xinru Gao, Yang Yang, Xin Bian, Bing Wang, Xiaofei Liu, Yan Wang, Dan Su, Guang Zhang, Lizhe Qu, Na Zhang

**Affiliations:** ^1^ Harbin University of Commerce, Harbin, Heilongjiang, China; ^2^ Shanghai Municipal Hospital of Traditional Chinese Medicine, Shanghai, China

**Keywords:** SPI-SA interpenetrating network hydrogel scaffold, mechanical properties, structure, three-dimensional cell culture, bio-compatibility, adhesion ability

## Abstract

Cell-cultured meat, which is obtained by adsorbing cells on the three-dimensional scaffold, is considered a potential solution to animal welfare issues. Edible and safe cell-cultured meat scaffolds are a key part of its research. Soy protein isolate (SPI) hydrogel has a three-dimensional network structure and has been studied for L929 cell culture because of its non-toxicity and biocompatibility. However, the toughness and mechanical properties of SPI hydrogel are not enough to bear the requirements of cell cultivation. In this paper, sodium alginate (SA) was added to SPI hydrogel, and the interpenetrating network (IPN) technology was used to construct SPI-SA IPN hydrogel by transglutaminase and Ca^2+^ double crosslinking method. SPI-SA IPN hydrogel has excellent mechanical properties, structural stability and biodegradable performance than SPI hydrogel. The bio-compatibility and degradability of L929 and C2C12 cells on SPI-SA IPN hydrogel were studied by cytotoxicity, trypan blue and living/dead cell staining, and the growth law of the hydrogel as a scaffold for cell culture was analyzed. The results showed that L929/C2C12 cells can proliferate normally and adhere in hydrogel and have good bio-compatibility. L929 cells with size about 20–50 µm have better adhesion and growth abilities on SPI-SA IPN hydrogel than C2C12 cells with 100–300 µm. Therefore, the SPI-SA IPN hydrogel is non-toxic and supports the growth of cells in the pores of the material. This study provides a reference for the application of SPI-SA IPN hydrogels *in vitro* cell growth.

## 1 Introduction

**FIGURE 1:**
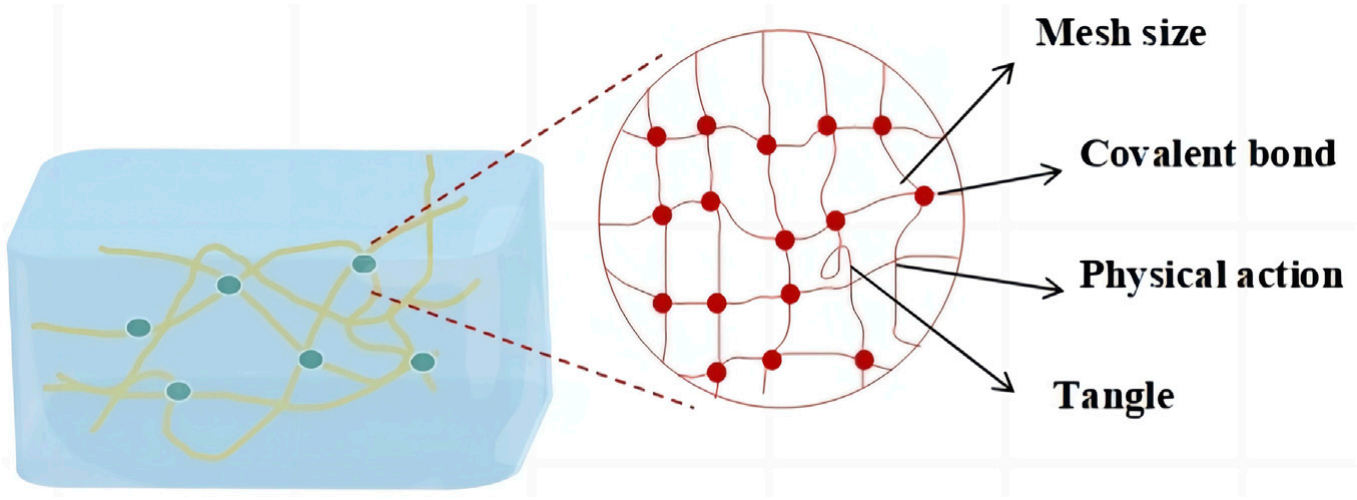
Three-dimensional network structure of natural polymer materialshydrogel.

In recent years, many studies have focused on the research of the cell-cultured meat among which the scaffold of three-dimensional cell culture is a key technology. The three-dimensional culture scaffold of cells can promote the growth, attachment and migration of cells, provide appropriate biochemical and biophysical clues, and support cells to exchange nutrients and oxygen in the scaffold, thus better simulating the micro-environment of living tissues. Thus, three-dimensional culture scaffolds need to meet a series of conditions, including bio-compatibility, reproducibility, high porosity (with specific pore size and inter-connectivity), suitable biodegradability, sufficient mechanical properties to support tissue growth and appropriate biochemical functions (Salerno et al., 2015). At present, common three-dimensional cell culture scaffolds include extracellular matrix scaffold, solid porous scaffold, fiber scaffold and hydrogel scaffold, which have improved cell culture technology (Vila-Parrondo et al., 2020). Among them, the hydrogel scaffold has a flexible and soft structure, and its physical and chemical properties are very close to the soft tissue in the organism, facilitating the exchange of nutrients and metabolic substances by cells, which makes hydrogel one of the most potential biomaterials for cell scaffolds *in vitro* (Zoratto and Matricardi, 2018). Hydrogels consist of a three-dimensional network of hydrophilic polymer chains (Figure 1), in which water is the dispersed phase and accounts for at least 70% of the gel weight (Tan and Joyner, 2020). The texture of hydrogel is between solid and liquid, which lead it has dual properties of solid and liquid (Kopeek and Yang, 2007). It is insoluble in water, but it has the ability to absorb a large amount of water because of its hydrophilic part. It can obviously absorb water and swell in water, and has a strong ability to retain water (Katyal et al., 2020). Hydrogels are formed by physical or chemical crosslinking of synthetic, natural or hybrid polymers, which have excellent mechanical properties, but poor bio-compatibility and adjustability, and have certain cytotoxicity and food insecurity (Ye et al., 2023). Therefore, the current related research is changing to natural polymer hydrogel (Brindha et al., 2019).

**FIGURE 2:**
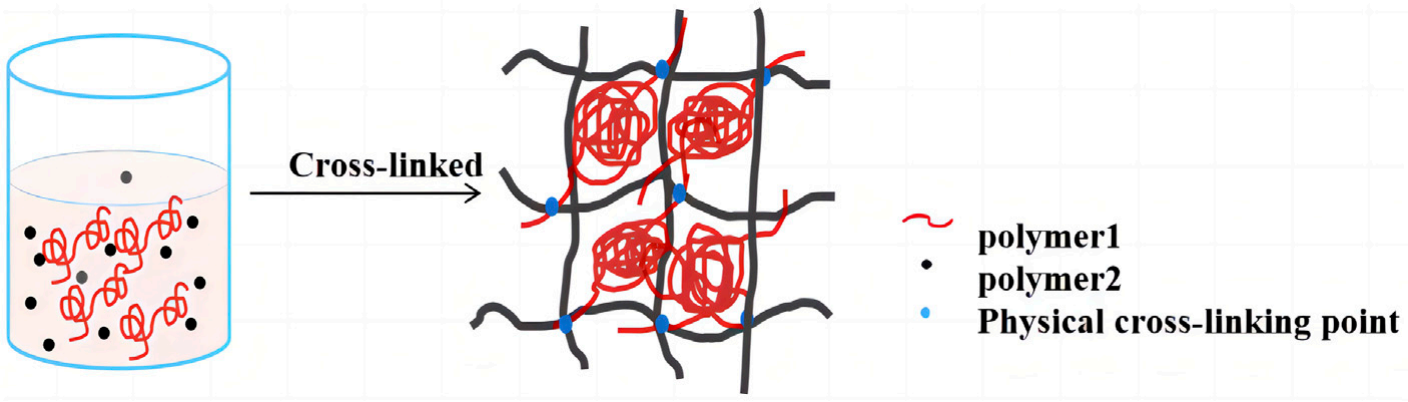
Schematic diagram of formation mechanism of IPN hydrogel.

Hydrogels of natural polymer materials usually come from polysaccharides or protein (Ghanbari et al., 2021a). In the development of hydrogels, protein has inherent advantages over polysaccharides (Ghanbari et al., 2022a). Protein contains many different amino acids, and many reactive groups can be used as sites for chemical modification and crosslinking to form polymer structures (Cuadri et al., 2016). Among all reported hydrogels, protein-based hydrogels have also been widely developed and studied by researchers because of their excellent characteristics, such as high nutritional value, bio-compatibility, biodegradability, adjustable mechanical properties and low toxicity compared with synthetic polymers (Farwa et al., 2022). Common protein hydrogel materials include collagen, silk fibroin, gelatin (Ghanbari et al., 2021b), *etc.*, but most of these proteins belong to animal proteins with high application costs, and because of their complex structure, their structural changes are often limited (Ghanbari et al., 2021c; Ghanbari et al., 2021d). In addition, compared to animal derived proteins, plant derived proteins may be safer because they have a lower likelihood of spreading zoonotic diseases (Surya et al., 2023). Soybean protein, as one of the most abundant sources of plant protein, has high nutritional value, environmental friendliness, and a wide range of sources, and has been widely used in the food industry (Liu et al., 2023). Soybean protein contains amino acids with polar functional groups that can undergo chemical reactions and are easily modified. However, it was found that the hydrogel prepared by single SPI has poor performance, and the hydrogel prepared by SPI with high gel concentration also showed poor water solubility (Dong et al., 2023). Therefore, interpenetrating network (IPN) technology is considered to improve the gel properties and solubility of SPI by adding polysaccharides to the system. IPN hydrogel is composed of two or more polymers, which are generally synthesized and interconnected by physical or chemical methods (Figure 2) (Chen et al., 2019). Among them, double network hydrogel is a special form of interpenetrating network hydrogel, which consists of two permeable polymer networks with unique characteristics, in which the rigid network is the first network and the flexible network is the second network (Wang and Wei, 2017). The formation of interpenetrating network can retain the characteristics of each network structure and improve the stability of materials due to interlocking structures in the cross-linked networks (Wu et al., 2023). Many IPN hydrogels are toxic and inedible (DingYB et al., 2022). At present, interpenetrating network hydrogels derived from natural materials have become the focus of researchers. It has been found that the IPN hydrogel formed by the synergistic effect of two or more biomacromolecules (such as protein and polysaccharide) has a highly entangled network, which can improve the mechanical properties of biopolymer hydrogel (Ng et al., 2023). Nowadays, IPN hydrogels are usually prepared from natural polysaccharides and protein (Le et al., 2017). Compared with hydrogels prepared by other cross-linking methods, hydrogels prepared by enzyme cross-linking are safe, edible and biocompatible. It has been studied to construct interpenetrating network hydrogel with natural polymers such as beet pectin, corn fibrin glue and chitosan and SPI to improve the functional characteristics of SPI hydrogel. For example, some scholars prepared soy protein isolate/beet pectin double-network hydrogels by heat treatment and laccase two-step gel method, and found that compared with single-network hydrogels, double-network hydrogels have excellent mechanical properties, water retention and microstructure (Zhu et al., 2016). Yan et al. developed a novel dual-network hydrogel of corn fibrin glue and SPI by laccase and gluconic acid δ-lactone (Yan et al., 2020). The dual-network hydrogel has pH response and high delivery and release efficiency, and can be used as an oral delivery tool for heat-sensitive bioactive compounds. TG enzyme can catalyze the formation of amide bond between glutamine and lysine residues in SPI, form the spatial structure of SPI and improve gel properties. Moreover, sodium alginate (SA) is a natural polysaccharide macromolecule composed of β-d-mannuronic acid and α-l-guluronic acid. It is a common thickener in food and has good gelling ability, which can be used to improve the toughness and viscoelasticity of hydrogels (Shen et al., 2020). However, the possibility of constructing IPN hydrogel using the synergistic effect of TG enzyme and SPI, calcium ion and SA, and their effect as cell scaffolds has not been studied yet.

L929 mouse fibroblast is one of the commonly used cells to explore the potential of hydrogel as a bionic extracellular matrix scaffold, and it is also a commonly used cell model to study the bio-compatibility of hydrogel (Govindan and Mohamed, 2023). Guan and co-authors prepared injectable gelatin/dextran oxide hydrogel for the treatment of acute skin injury (Guan et al., 2021). After L929 cells were cultured on the hydrogel in three dimensions, CCK8 staining with living/dead cells showed that the hydrogel with good cell compatibility provided a good microenvironment for the growth of cells. Guo et al. prepared *in situ* mechanically adjustable double cross-linking *in situ* mechanically adjustable double cross-linking hyaluronic acid/polylysine hydrogel. After L929 cells were cultured in three-dimensional hydrogel, it was found that the prepared hydrogel had enough culture space to make the hydrogel have good potential in biomaterial application and disease treatment (Guo et al., 2021). C2C12 mouse myoblast is the precursor cell of skeletal muscle, which is a kind of myogenic cell located on the surface of mature muscle fiber under normal conditions, and has strong proliferation and differentiation ability (Brezar et al., 2023). C2C12 cell is the first choice model for studying the proliferation and differentiation of myoblast *in vitro*. Some researchers used mouse myoblast cell line (C2C12) to evaluate the applicability of pigskin gel in skeletal muscle tissue engineering, and showed good research results (Xu et al., 2022). Elkhoury et al. studied the possibility of fish gelatin methacryloyl hydrogel as a scaffold for C2C12 myoblasts, and found that C2C12 cells cultured with this hydrogel showed good bio-compatibility, and cell proliferation (Elkhoury et al., 2021).

Therefore, SPI-SA-IPN hydrogel scaffold was prepared in this study, and L929 and C2C12 cells with different sizes were selected and compared for three-dimensional culture in the scaffold for 1, 3, 5, and 7 days. The rheological properties and structure of prepared SPI-SA-IPN hydrogel scaffold were detected. The biodegradability, bio-compatibility, cell adhesion ability, and biological toxicity of the scaffold at were evaluated, providing a theoretical basis for further research on three-dimensional cell culture scaffolds.

## 2 Materials and methods

### 2.1 Materials

Soybean protein isolate (950.0 g/kg protein content, dry basis) was purchased from Shandong Yu Wang Ecology Food Industry Co., Ltd (Shandong, China) to prepare SPI-SA IPN hydrogels. Sodium alginate was brought from He’nan tanggui foods Co., Ltd (Henan, China) to prepare SPI-SA IPN hydrogels. PBS buffer solution was purchased from Xiamen haibiao technology Co., Ltd (Xiamen, China) to be used for washing cells. CCK-8 kit was bought from Jiangsu biyuntian biology technology company (Jiangsu, China) to determine the cytotoxicity of biomaterials. Sterile PBS was purchased from Beijing Bo’ao Tuoda technology Co., Ltd (Beijing, China) to be used for soaking materials. Live/Dead cell staining, dimethyl sulfoxide (DMSO), trypan blue, minimum essential medium (MEM) culture medium were all bought from Beijing Solarbio science and technology Co., Ltd (Beijing, China) to observe by fluorescent staining. Fetal calf serum was purchased from Hangzhou sijiqing biological engineering Co., Ltd (Hangzhou, China) to prepare complete culture medium. All other reagents were analytical grade.

### 2.2 Preparation of SPI-SA IPN hydrogel scaffold

SPI-SA IPN hydrogel scaffold was prepared according to the previous pre-experiment and the method of Zhong with slightly modifications (Zhong et al., 2021). SPI and sodium alginate powder were fully dissolved and mixed in distilled water to obtain the both final concentration of 70 g/L. The TG enzyme powder was added into the mixed solution with 40 U/g. Calcium chloride was dissolved in distilled water to prepare 15 mmol/L CaCl_2_ solution. Adding the CaCl_2_ solution into TG-SPI/SA solution drop by drop with stirring to form pre-reaction solution. The pre-reaction solution was placed in a water bath at 50°C for 2 h. After the reaction, the enzyme was inactivated at 90°C for 5 min. And then, the whole system was cooled to room temperature to obtain SPI-SA IPN hydrogel scaffold. The SPI hydrogel prepared by SPI solution and TG enzyme reaction was used as a control. And the prepared hydrogels were stored at 4°C before using.

### 2.3 Determination of mechanical properties of SPI-SA IPN hydrogel scaffold

The gel hardness of hydrogel was determined according to the method of Wu et al. (Xiao et al., 2019). The P 0.5 probe was used, the compression ratio is 50%, the pre-test, test and post-test rates are 1.0 mm/s, the trigger force is 5.0 g, and the interval between two measurements is 3 s, and each treatment group repeats 3 times. Firstly, strain-related measurements were made to obtain the linear viscoelastic region of the hydrogel samples. After the strain-related measurement, a frequencyrelated measurement was made at a fixed strain of 0.628–62.8 rad s^−1^ at 25°C.

The determination of the dynamic rheological properties of hydrogel refers to the method of Zhong et al. (Zhong et al., 2021). The dynamic rheological properties of the hydrogels were measured using a shear rheometer (MCR302, Anton Paar, Austria) fitted with parallel plates (50 mm diameter and 1 mm gap).

### 2.4 Observation on structure of SPI-SA IPN hydrogel scaffold

The macro morphology of SPI hydrogel and SPI-SA IPN hydrogel were observed, and the microstructure of hydrogels was determined by scanning electron microscope according to Xiao et al. (Changling et al., 2021) Briefly, the hydrogel samples were subjected to freeze-drying and cryo-fractured in liquid nitrogen with a scalpel to expose the fracture surfaces. A JEOL JSM 7800F field emission scanning electron microscope operated at an accelerating voltage of 5 kV was used to observe the gold-coated sample cross-sections.

### 2.5 Determination of biodegradability of hydrogels

The biodegradability of SPI-SA IPN hydrogel was determined according to the method of Burke (Burke et al., 2019) with slightly modifications. The hydrogel was soaked in PBS containing 10,000 U/mL lysozyme overnight until the swelling balance was reached, and its weight was determined to be mr. The samples were incubated at 37°C and the degradation solution was changed every 2 days. The hydrogel was take out from PBS, rinsed with deionized water, wiped off the surface moisture. And then, the hydrogel was dried at room temperature for 5 min, and its quality was determined. The weight of the hydrogel at 1, 3, 5, 7 days was measured, respectively, which was set as m_t_. The biodegradation rate of hydrogel scaffold materials can be calculated by Eq. 1.

$$\text{Degradation rate}\left(\%\right)=\frac{m_{t}-m_{r}}{m_{t}}$$ (1)

### 2.6 Cell culture and morphological observation

#### 2.6.1 Cell culture of L929 and C2C12 on SPI-SA IPN hydrogel scaffold

L929 and C2C12 cells were seeded into 24-well plates at 5×10^4^ cells/well. The scaffold material applied to cell culture was prepared at the other 24-well plate with a cylindrical sheet gel with a bottom area of approximately 14 mm and a height of 2 mm (Yameen et al., 2023). The prepared hydrogels were freeze-dried in a freezer (SCIENTZ-12N, Ningbo Xinzhi Biotechnology Co., Ltd., Ningbo, China), the hydrogel samples were placed in a 24-well plate, soaked in 75% ethanol for 2 h, and sterilized by ultraviolet irradiation for 2 h. Then sterile PBS was added to make it swell, and then PBS was removed to soak the material with culture solution. After 6 h, the culture solution was aspirated and the L929 or C2C12 cell suspension of 500 µL was inoculated onto the material in each well (Dong et al., 2023). The well plates were incubated at 37°C in a 5% CO_2_ incubator (HCP-168, Qingdao Haier biomedical Co., Ltd., Qingdao, China), and the culture medium was changed every other day (Monteiro et al., 2023).

#### 2.6.2 Morphology of L929 and C2C12 cells

The morphology of L929 and C2C12 cells in the normal culture state was photographed using an inverted microscope (WYS-41XDY, Tianjin Weiyi Optical Instrument Co., Ltd., Tianjin, China) according to previous methods (Huang et al., 2022). The magnification of inverted microscope is ×200 to observe the cell morphology.

### 2.7 Determination of cell bio-compatibility

#### 2.7.1 Determination of cell proliferation activity

The cell proliferation activity of L929 cells and C2C12 cells cultured in SPI-SA IPN hydrogel scaffold for 1, 3, 5, and 7 days was determined by CCK-8 kit according to the method of Xiao et al. with slight modifications (Xiao et al., 2019). The L929 and C2C12 cells were cultured on SPI-SA IPN hydrogel scaffold according to the method of 2.5.1. After 1, 3, 5, 7 days of culture, L929 and C2C12 cells were cleaned with sterile PBS for 2–3 times after absorbing the original culture medium, and fresh culture medium containing 10% CCK-8 reagent was added to each well in equal amount, and then it was incubated in an incubator at 37°C for 3 h. Then suck 100 µL of solution from each well into a new 96-well plate. After gently shaking for 5 min, the OD value at 450 nm was detected by microplate reader (M2e, Meigu molecular instrument Co., Ltd., Shanghai, China). The results were collected from three parallel wells, and cell proliferation activities were respected by optical density at 450 nm.

#### 2.7.2 Determination of living/dead cells on SPI-SA IPN hydrogels

Live cells on SPI-SA IPN hydrogels were observed by the experiment of live/dead cell staining (Yang et al., 2019). L929 or C2C12 cells (10^5^ cells per well) were inoculated in 24-well culture plates and cells were cultured at 37°C in 5% CO_2_ for 3 days (cell clusters on hydrogel were found in the third day by pre-experiments). Subsequently, the cell culture medium was removed and the 24-well hydrogels were rinsed with PBS. A 100 μL/well calcein AM/PI solution was added to the 24-well hydrogel plate and incubated in the dark for 30 min. After rinsing with PBS three times before imaging, the hydrogel plate was placed under a confocal microscope (WYS-41XDY, Tianjin Weiyi Optical Instrument Co., Ltd., Tianjin, China). The live cells showed green fluorescence, while the dead cells showed red fluorescence. Live cells were observed by live/dead staining of L929 and C2C12 cells on SPI-SA IPN hydrogels for fluorescent staining observation with a magnification of ×200 (Orr et al., 2021).

### 2.8 Determination of cell adhesion ability on SPI-SA IPN hydrogels

In order to evaluate the adhesion of the 2 cells on the hydrogel, L929 and C2C12 cells were cultured in SPI-SA IPN hydrogel for 1, 3, 5, and 7 days, and then stained with trypan blue. Referring to the method of Yu et al. (Yu et al., 2019), the cells were incubated on the scaffold for 5 days, the medium was aspirated, and the material in each well was transferred to a new well with sterile forceps, rinsed three times with PBS, and 500 μL of 0.25% trypsin was added to each piece of material. The dead cells were stained with a distinct color of blue, while the live cells remained colorless and transparent. The magnification of the images were ×100.

### 2.9 Determination of cytotoxicity experiments

According to the international standard ISO 10993, the cytotoxicity of biomaterials was determined by CCK-8 kit (Shanmugam et al., 2023). The SPI-SA IPN hydrogel with the best ratio was fully sterilized by ultraviolet for 1 h, then completely soaked in complete culture medium, and leached in a water bath at 37°C for 24 h to obtain the leaching solution. Filter and sterilize, and store at 4°C for later use.

Inoculate the cultured cells into 100 μL to 96-well plates, with about 1,000 cells in each well. The cells are spread on the bottom of the well by a single layer. After 24 h of culture, the cells completely adhere to the wall, discard the original culture medium, add 100 μL hydrogel extract or fresh culture medium, and continue to culture in a CO_2_ incubator with a volume fraction of 5% at 37°C (Amin et al., 2023). The complete medium (no cells) was set as the blank group, a complete medium with cells was set as control group, and then the hydrogel extract was added as the experimental group. Five wells were set in each group, and the plates were removed at 1, 3, 5 and 7 days, respectively, and the cell viability was determined by CCK-8 method. The relative growth rate (RGR, %) was calculated according to (Eq. 2) and the cytotoxicity of the scaffolds was evaluated.

$$\text{RGR}\left(\%\right)=\frac{{\text{OD}}_{\text{experimental}\;\text{group}}-{\text{OD}}_{\text{blank}\;\text{group}}}{{\text{OD}}_{\text{contrast}\;\text{group}}-{\text{OD}}_{\text{blank}\;\text{group}}}\times 100$$ (2)

### 2.10 Statistical analysis

All experiments were conducted at least five times in parallel each time, and the results were expressed as mean ± standard deviation. The data obtained from the experiments were analyzed by ANOVA using Statistical Package for Social Science (SPSS 20.0) and the differences were considered significant when *p* < 0.05; Origin 8.5 software was used for graphing.

## 3 Results and discussion

### 3.1 Analysis of mechanical properties of SPI-SA IPN hydrogel

**FIGURE 3:**
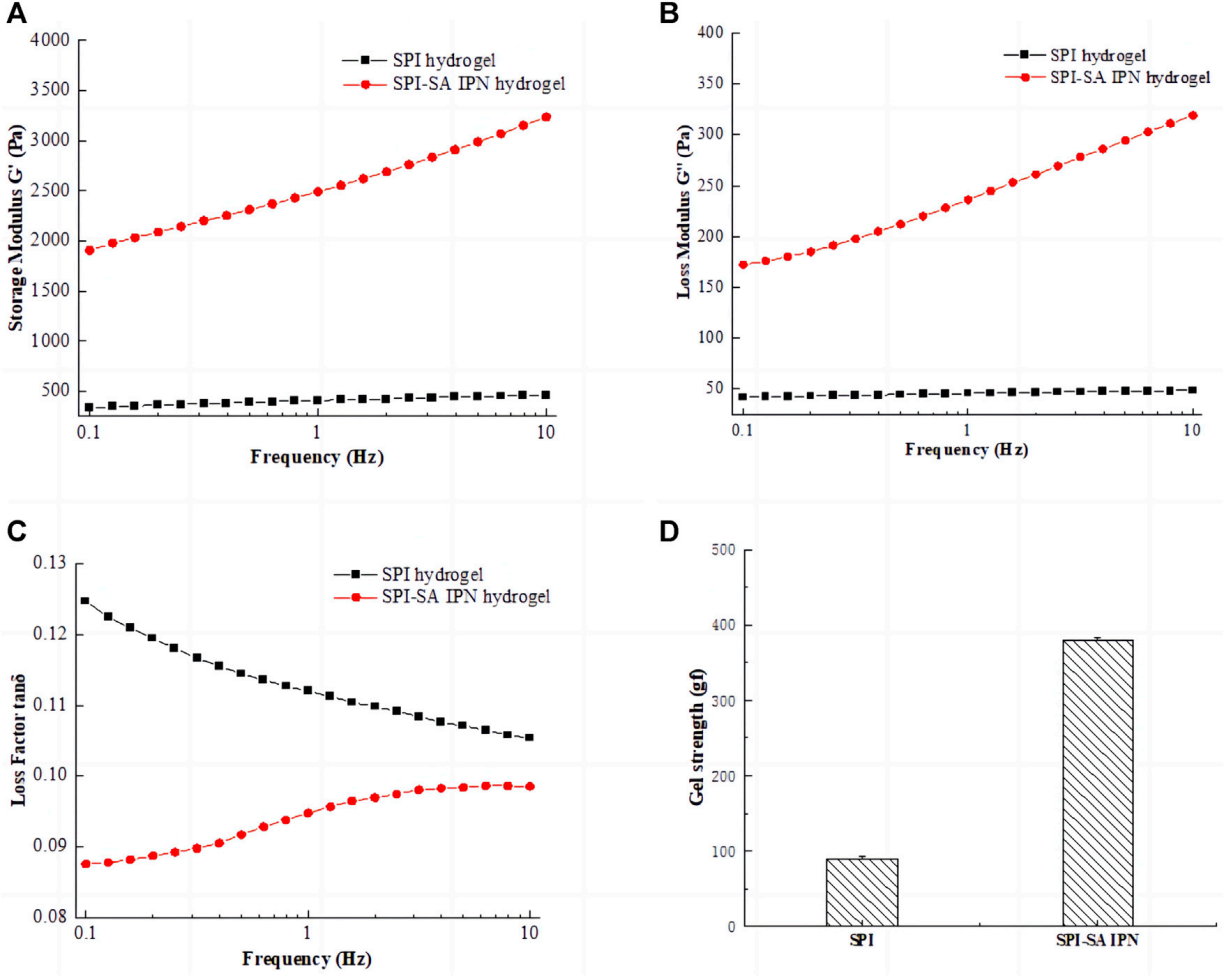
Rheological properties of SPI hydrogel and SPI-SA IPN: **(A)** storage modulus G'; **(B)** loss modulus G"; **(C)** loss factor tan δ; **(D)** gel strength.

Rheological properties and gel strength were used to reflect the mechanical properties of SPI hydrogels and SPI-SA IPN hydrogel. The storage modulus G′, loss modulus G “and loss factor tanδ of SPI hydrogel and SPI-SA IPN hydrogel were affected by the change of frequency (0.1–10 Hz), and the gel strength of hydrogels under the same deformation force was shown in Figure 3. The G′ and G″of hydrogel increased with the increase of frequency in a certain range, which indicated that hydrogel will be affected by external force (Figures 3A,B). The storage modulus (G′) and loss modulus (G″) showed the elasticity and viscosity characteristics of the hydrogel, respectively. In the whole frequency range, the G′ and G″ values of SPI-SA IPN hydrogel were much higher than those of SPI hydrogel, which indicated that the viscoelasticity of SPI-SA IPN hydrogel has been greatly improved with the construction of IPN hydrogel, and IPN technology has improved the mechanical properties of single material hydrogel. This is similar to the result of Cao et al. found that the rheological property of guar gum/gellan gum interpenetrating network hydrogel is much higher than that of single guar gum gel (Cao et al., 2021).

The variation curve of loss factor with frequency (0.1–10 Hz) was shown in Figure 3C. Loss factor refers to the tangent of the phase difference angle between strain and stress period of viscoelastic materials under the action of alternating force field, and it is also equal to the ratio of loss modulus to storage modulus of this material, that is, tanδ = G"/G'. The magnitude of the loss factor represents the viscoelastic properties of the material. The larger the loss factor, the greater viscosity of the material, and the smaller loss factor, the greater the elasticity of the material. The state of materials can be expressed within a certain range. The results showed that the tan δ of SPI and SPI-SA IPN hydrogels was far less than 1. This shown that all hydrogels have stable structure and good viscoelasticity.

The gel strength of SPI hydrogel and SPI-SA IPN hydrogel was shown in Figure 3D. Compared with single SPI hydrogel, IPN hydrogel showed higher gel hardness, and the gel strength of SPI-SA IPN hydrogel was nearly four times higher than that of SPI hydrogel. When compressed at 50% strain, the optimized SPI-SA IPN hydrogel showed structural integrity and higher compressive stress. In contrast, the structure of pure SPI hydrogel is easy to be destroyed. This result was also consistent with the analysis of rheological properties, which shown that the construction of IPN hydrogel can greatly improve the mechanical properties of hydrogel (Valipour et al., 2023). Ghanbari and co-authors have developed a group of injectable hydrogels comprised of oxidized alginate/gelatin, and the hydrogel was strengthened by the amount of Zn_2_SiO_4_ nanoparticles. The research results indicated that Zn_2_SiO_4_ improved the mechanical characteristics of the hydrogels. Therefore, the preparation method of hydrogel scaffold has a great impact on the mechanical properties of hydrogel (Ghanbari et al., 2022b).

### 3.2 Analysis of structure of SPI-SA IPN hydrogel

**FIGURE 4:**
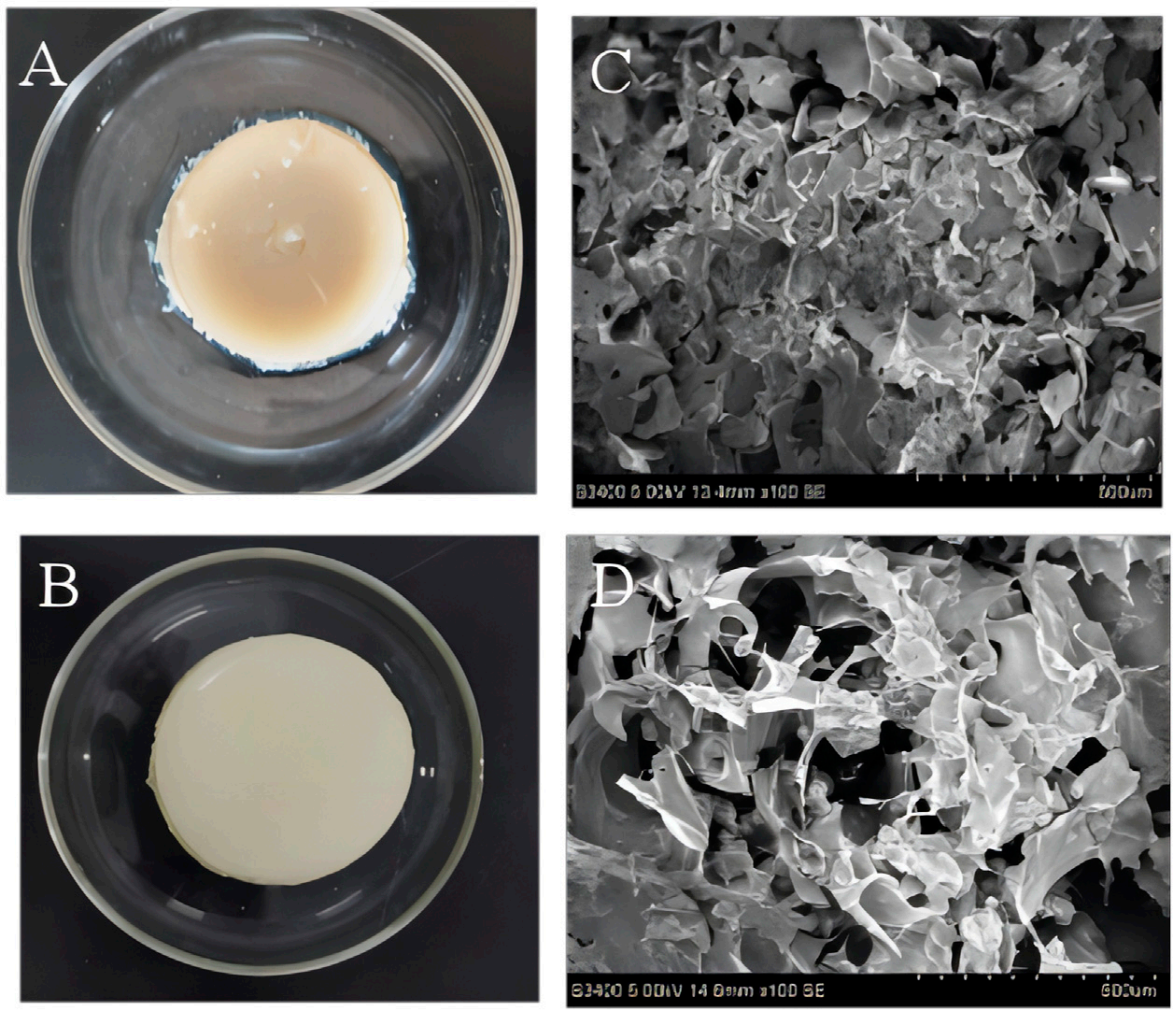
Macroscopic structure **(A, B)** and microstructure **(C, D)** images of the SPI hydrogel **(A, C)** and SPI-SA interpenetrating hydrogel **(B, D)**.

The microstructure of SPI hydrogel and SPI-SA IPN hydrogel were observed macroscopically and photographed by scanning electron microscope (Figure 4). It can be observed from Figure 4 that both SPI hydrogel (Figure 4A) and SPI-SA IPN hydrogel (Figure 4B) can form gel. However, SPI hydrogel formed by SPI and TG enzyme has unstable structure, and the formed SPI hydrogel is relatively soft. After being placed for a period of time, the gel is separeted by water with a slight collapse in the middle. The structure of SPI-SA IPN hydrogel is more flexible and elastic. The microscopic images of SPI hydrogel obtained by scanning electron microscope (Figure 4C) showed that there is no obvious pore structure on the surface of hydrogel, which is not suitable for cell adhesion. However, the microscopic image of SPI-SA IPN hydrogel (Figure 4D) showed that there are pores of different sizes on the surface of the hydrogel with pore diameters between 100–300 µ m, and the surface is rough. The 3D network structure with specific pore morphology is very important for cell growth. SPI-SA IPN hydrogel showed lamellar distribution, and the macropores on each layer are interlaced with the interpenetration of macropores and micropores, which is suitable for cell adhesion and transportation of cell nutrient, and promotes cell growth (Zhong et al., 2021).

### 3.4 Degradation analysis of SPI-SA IPN hydrogel

**FIGURE 5:**
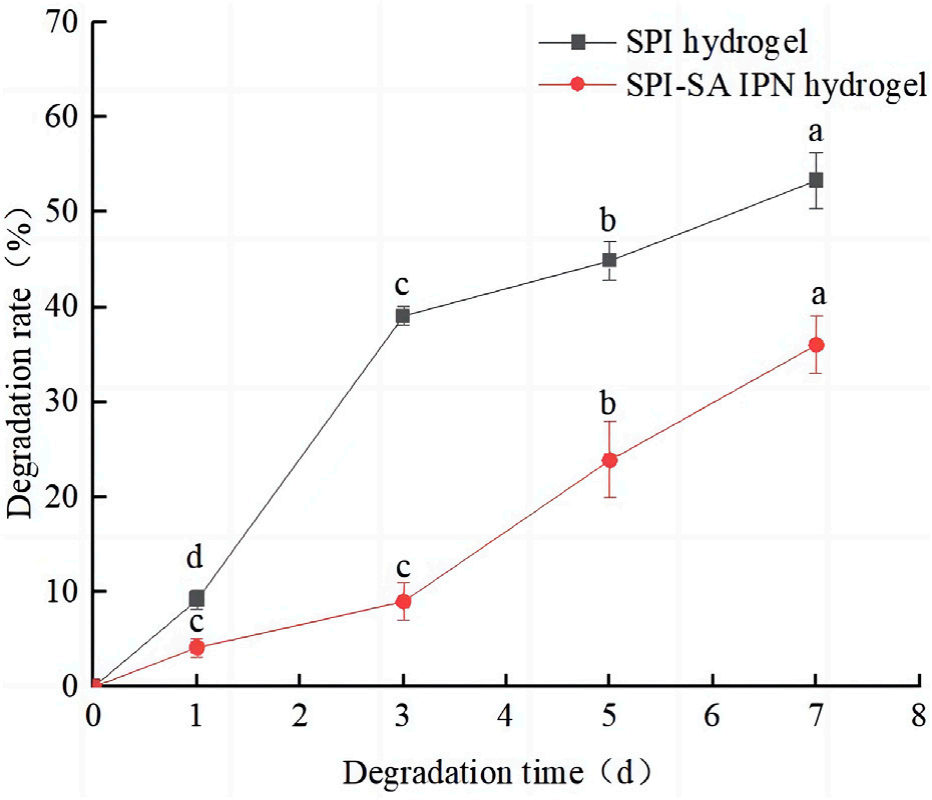
The degradation rates of the hydrogels. The black line represents SPI hydrogel; The red line represents SPI-SA IPN hydrogel. Different letters (a–d) over a bar indicate significant differences (*p* < 0.05).

In order to evaluate the potential changes of cells on the hydrogel after inoculation, the degradation rates of SPI-SA IPN hydrogel in simulated physiological solution (PBS- lysozyme solution) for 1, 3, 5 and 7 days were measured. SPI hydrogel was used as control group and the results were shown in Figure 5. With the increase of degradation days of SPI and SPI-SA IPN hydrogel, the degradation rates of two hydrogel scaffolds were increased significantly (*p* < 0.05), and the mass loss of SPI hydrogel was 9.2% ± 0.9% within 24 h and 53.3% ± 3.9% within 7 days, which was mainly due to the hydrophilicity of SPI and the unstable structure of single SPI hydrogel. On the seventh day, the mass loss of SPI-SA IPN hydrogel scaffold was 36.0% ± 3.3%, which was significantly decreased compared with the degradation rate of SPI hydrogel. The difference in mass loss between the two hydrogels was due to the ionic interaction between SA and calcium in the preparation of SPI-SA IPN hydrogel to support the hydrogel scaffold, which led to the relatively difficult degradation of IPN hydrogel. However, IPN hydrogel will form a wet layer around the surface after water absorption and swelling, which will be degraded by lysozyme. This cycle continues layer by layer, slowing down the degradation process of IPN. Badhe et al. studied the degradability of gelatin and chitosan double-layer scaffold, and found that the degradability of double-layer scaffold was better than that of single-layer hydrophilic material, which was similar to the results of this study (Badhe et al., 2017). The degradation performance of hydrogel scaffold is an important issue in tissue engineering application. The ideal hydrogel scaffold should adjust the degradation speed of scaffold according to the regeneration process of cell tissue and support the formation of new tissue (Qi et al., 2019). Previous studies have found that the degradation of scaffold lasts longer and can support cell proliferation and full cellularization of scaffold, so SPI-SA IPN hydrogel with longer degradation time is more likely to support cells in it than SPI hydrogel (Haleem and Chu, 2010). Therefore, SPI-SA IPN hydrogel was used for subsequent three-dimensional culture of cells.

### 3.5 Morphogram of L929 and C2C12 cells

**FIGURE 6:**
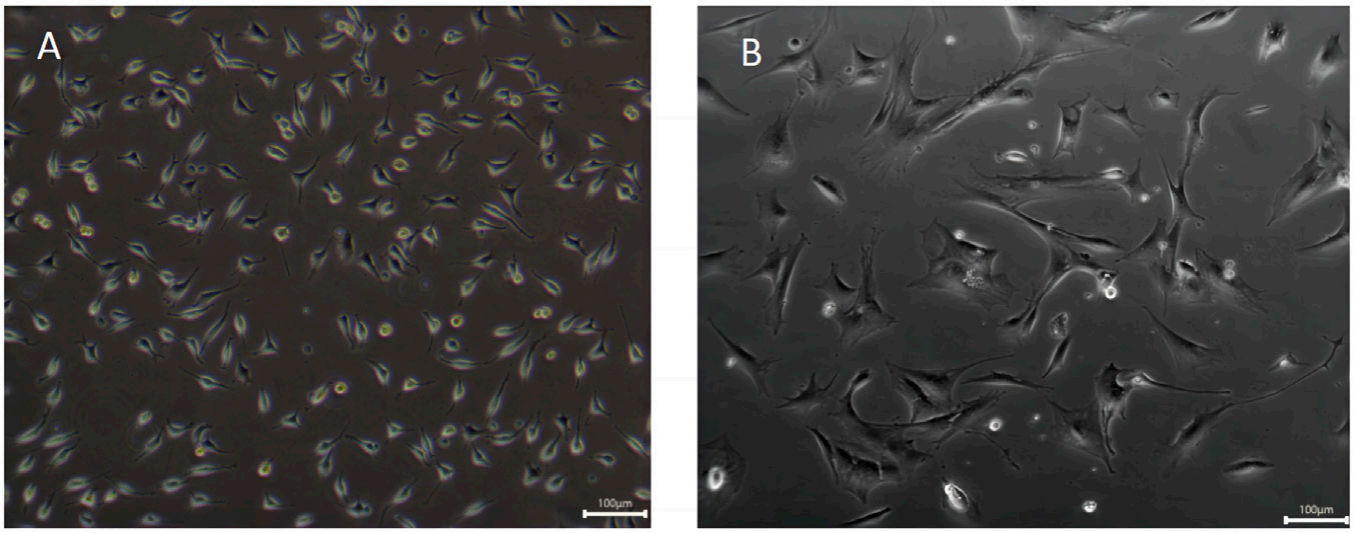
Morphology observation of fibroblasts L929 cells **(A)** and myoblasts C2C12 cells **(B)** with ×200 magnification.

The morphology of L929 mouse fibroblasts and C2C12 mouse myogenic cells grown in culture flasks was observed using an inverted microscope, and the results were shown in Figures 6A,B. L929 murine fibroblasts grew in the culture flask in an apposed wall with a spindle or flat star-shaped cell growth morphology with protrusions, and determination of cell size revealed that the size of L929 cells was mainly concentrated in the range of 20–50 µm (Figure 6A) (Bauer et al., 2013). Moreover, C2C12 mouse myogenic cells have a fibroblast-like morphology, mainly spindle-shaped, and it has been reported that C2C12 cells are initially mononucleated and spindle-forming myoblasts, followed by cell fusion to form multinucleated structures, and the cell size was measured between 100 and 300 µm (Figure 6B) (Li et al., 2022). Savaris found that limiting the ideal pore size of scaffold materials to 100–300 μm is more suitable for supporting the potential application of fibroblast growth (40–150 μm) and cell regeneration (Savaris et al., 2019). These shown that L929 mouse fibroblasts and C2C12 mouse myogenic cells are two types of cells with differing growth morphology and size, which can be used to compare the effect of cells with different sizes on three-dimensional growth within SPI-SA IPN hydrogels in subsequent experiments.

### 3.6 Analysis of cell bio-compatibility inside the hydrogel scaffold

**FIGURE 7:**
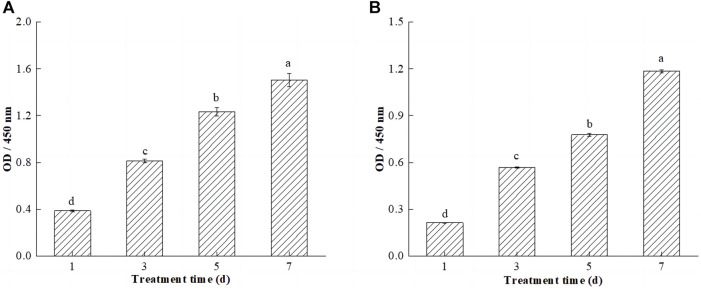
Efects of SPI-SA IPN hydrogel on proliferation of L929 cells **(A)** and C2C12 cells **(B)** at 1, 3, 5, and 7 days (×200). Different letters (a–d) over a bar indicated significant differences (*p* < 0.05).

The internal structure of hydrogels is similar to extracellular matrix, and in general, hydrogel-like bio-scaffolds with good bio-compatibility can allow cells to grow and proliferate in the scaffold network by adhesion. The effects of SPI-SA IPN hydrogels on the proliferation and viability of L929 and C2C12 cells at 1, 3, 5, and 7 days were investigated by CCK8 and live/dead cell staining to determine the bio-compatibility of the hydrogels, and the experimental results were shown in Figures 7A,B.

CCK-8 is a monosodium salt of tetrazolium that can be reduced to orange water-soluble methanamine by mitochondrial dehydrogenation in living cells. Dead or damaged cells do not show any dehydrogenase activity and do not exhibit color changes. There is a linear relationship between the shade of the reaction color and the number of living cells; if there are more living cells and the cell viability is greater, the color is darker, and if the cell activity is weaker, the color is less intense, so this technique can be used to estimate the percentage of living cells and to evaluate cell viability and proliferation capacity (Ding et al., 2021). The cell viability of L929 and C2C12 cells on hydrogels increased significantly (*p* < 0.05) with the increase of incubation time of the cells on hydrogels (Figures 7A,B). This is due to the ability of the SPI-SA-IPN hydrogel scaffold to provide a larger three-dimensional space for cell growth, which promotes the three-dimensional growth of cells. Elkhoury et al. studied the possibility of fish gelatin methacryloyl hydrogel as a scaffold for C2C12 myoblasts, and found that C2C12 cells cultured with this hydrogel showed good biocompatibility, and cell proliferation and proliferation were observed in the hydrogel (Elkhoury et al., 2021). Notably, comparing the proliferation activities of both cells on the hydrogel revealed that the proliferation activities of L929 cells was significantly higher than those of C2C12 cells. This may be due to the pore size of the hydrogel ranging from 100 to 300 μm, which provides a smaller spatial environment for C2C12 cells and slows down cell growth and proliferation.

**FIGURE 8:**
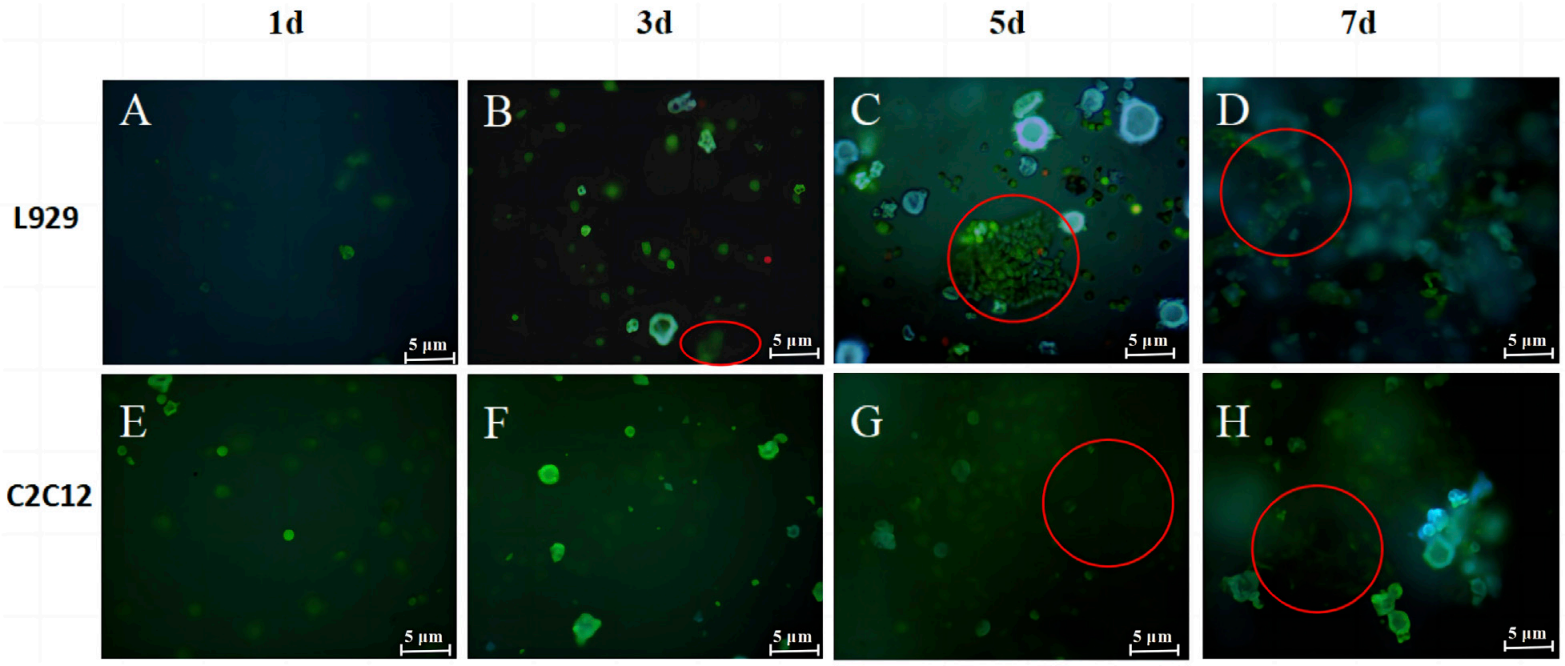
Live/death staining images for L929 **(A–D)** and C2C12 **(E–H)** cells in SPI-SA IPN hydrogels at 1, 3, 5 and 7 days with ×200 magnification.

The live/dead cell staining was used after a period of incubation to observe the growth status and distribution of cells to further determine the cytocompatibility of the hydrogel. The live/dead staining pictures of L929 in hydrogel after 1, 3, 5 and 7 days of culture were shown in Figures 8A–D. It can be seen that very few L929 morphologies are round, and most of the L929 morphologies have a shuttle shape adhering to the network and growing in a three-dimensional network distribution at day 1. The pictures on day 3 and 5 showed a further increase in green cell density and on day 3 L929 cells in the hydrogel began to undergo cell clustering, which increased as the cells grew inside the gel for longer periods of time (Figures 8B,C). After 7 days of culture, the live cells on the hydrogel were tightly connected and clustered together to form large cell clusters, and multilayered cell growth occurred (Figure 8D). These results are similar to the results of other studies on 3D cell scaffolds, such as the culture of RCCS cells on polyvinyl alcohol-hyaluronic acid-collagen macroporous hydrogels by Xie, which found that the cells, after attaching to the hydrogels, divide heavily to grow in clusters and secrete a lot of cell matrix thus adhering to the pore walls Wong (Bauer et al., 2013). This proved that L929 cells show three-dimensional growth on hydrogels. And the appropriate pore size and porosity for the cells allowed the smooth flow of nutrients in the gel, ensuring that the cells could grow horizontally and vertically in multiple dimensions.

The live/dead staining pictures of L929 in hydrogel after 1, 3, 5 and 7 days of culture were shown in Figure 8 It can be seen that most of the C2C12 morphology is round at day 1 (Figure 8E), and the C2C12 morphology is shuttle-shaped adhering to the network and growing and distributed in a three-dimensional network at day 3 (Figure 8F). After day 5, multiple layers of C2C12 were observed overlapping each other in the field of view and the cells proliferated further in the hydrogel (Figures 8G,H). And most of the cells observed at all time points were green live cells with very few red dead cells, which were only sporadically present among the large number of live cells. The co-culture illustrated that the cells are able to coexist with the hydrogel and grow well on the hydrogel. The staining results of live/dead cells were consistent with the results of cell proliferation, both proving that the scaffold has good bio-compatibility with L929 and C2C12 cells.

### 3.7 Analysis of the cell adhesion ability inside the hydrogel scaffold

**FIGURE 9:**
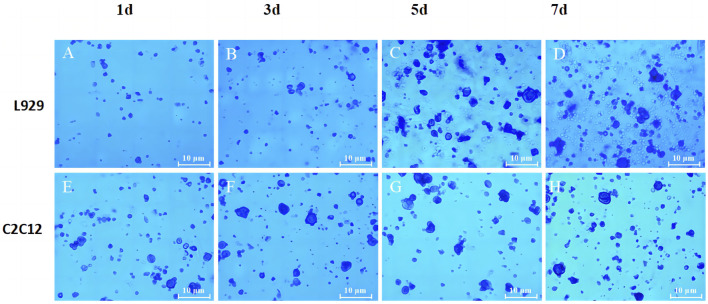
The trypan blue staining images of L929 **(A–D)** and C2C12 cells **(E–H)** on SPI-SA IPN hydrogel for 1, 3, 5 and 7 days of culture with ×100 magnification.

To better assess the adhesion ability of both cells on the SPI-SA IPN hydrogel, L929 cells and C2C12 cells were incubated in SPI-SA-IPN hydrogel for 1, 3, 5 and 7 days, and then stained with Taipan blue (Figure 9).

It can be seen that the number of live cells adhered to the hydrogel by trypsin digestion increased with the increase of culture time in the hydrogel (Figures 9A–D), indicating that the porous and relatively rough surface of the scaffold provided attachment sites for L929 cells after inoculation into the SPI-SA IPN hydrogel scaffold, and L929 cells underwent adhesion and proliferation in the hydrogel. When the L929 cells were inoculated into the hydrogel scaffold for 1 day, the number of live cells digested in the hydrogel scaffold was low, which may be due to the transfer of the cells from the wall culture to the three-dimensional culture in the scaffold, and the inoculated cells were not yet fully attached. When the cells were cultured in the hydrogel scaffold for 5 days and 7 days, the number of digested live cells increased significantly, which indicated that L929 cells inoculated into SPI-SA IPN hydrogel could grow and proliferate normally in the hydrogel scaffold after a period of attachment, confirming that L929 cells have better adhesion ability in SPI-SA IPN hydrogel. These results were consistent with other researches that composite hydrogels improved cell adhesion, which confirmed by MTT assay and cell adhesion study (Ghanbari et al., 2021c).

The number of live cells inside the hydrogel scaffold also increased with the increase of culture time after C2C12 cells were cultured in the hydrogel for 1, 3, 5, and 7 days (Figures 9E–H). This indicated that C2C12 cells can adhere to the SPI-SA IPN hydrogel and proliferate with the increase of culture time, but it can be observed from Figure 9H that the number of live cells was low after 7 days of culture in the hydrogel, which is not consistent with the growth and proliferation rate of C2C12 cells. These may be due to the size of C2C12 cells and the size of the SPI-SA IPN hydrogel. The pore size range was approximately the same, and after C2C12 was inoculated into the hydrogel scaffold, only a smaller number of cells could attach and grow inside the hydrogel scaffold, and after a period of growth, the cell growth and proliferation was inhibited due to the lack of space for C2C12 cells to grow inside the SPI-SA IPN hydrogel scaffold. Jiang et al. (Jiang et al., 2023) study a novel bioadhesive and antibacterial hydrogel composed of hydrophobically modified gelatin, oxidized konjac glucomannan and dopamine. This functional hydrogel has developed stability and strong tissue adhesion in liquid environment, even much higher than the adhesion of commercial fibrin glue to wounds.

Cell adhesion is a complex process influenced by a variety of factors, including cell behavior, biomaterial surface properties and environmental factors, and as it relates to scaffold surface properties, roughness, hydrophobicity, surface tension, chemical composition, porosity and pore size are key factors in cellular response. Bauer et al. (Haleem and Chu, 2010) Savaris found that limiting the ideal pore size of scaffold materials to 100–300 μm range is more suitable for potential applications in supporting fibroblast growth (40–150 μm) and cell regeneration. Garcia et al. (Savaris et al., 2019) Considering the size of both cell types, the growth status of L929 fibroblasts with cell size of 20–50 µm was better when the cells were cultured within the SPI-SA IPN hydrogel scaffold with a pore size of 100–300 μm, i.e., both L929 and C2C12 cells were able to undergo adhesion and proliferation on the hydrogel, but L929 cells on the SPI-SA IPN hydrogel had better adhesion and growth-promoting ability (Figures 9A–D) significantly higher than C2C12 cells (Figures 9E–H), and this result was corroborated with the results of cell viability within the SPI-SA IPN hydrogel scaffold for both cells.

### 3.8 Cytotoxicity analysis of SPI-SA IPN scaffolds

**FIGURE 10:**
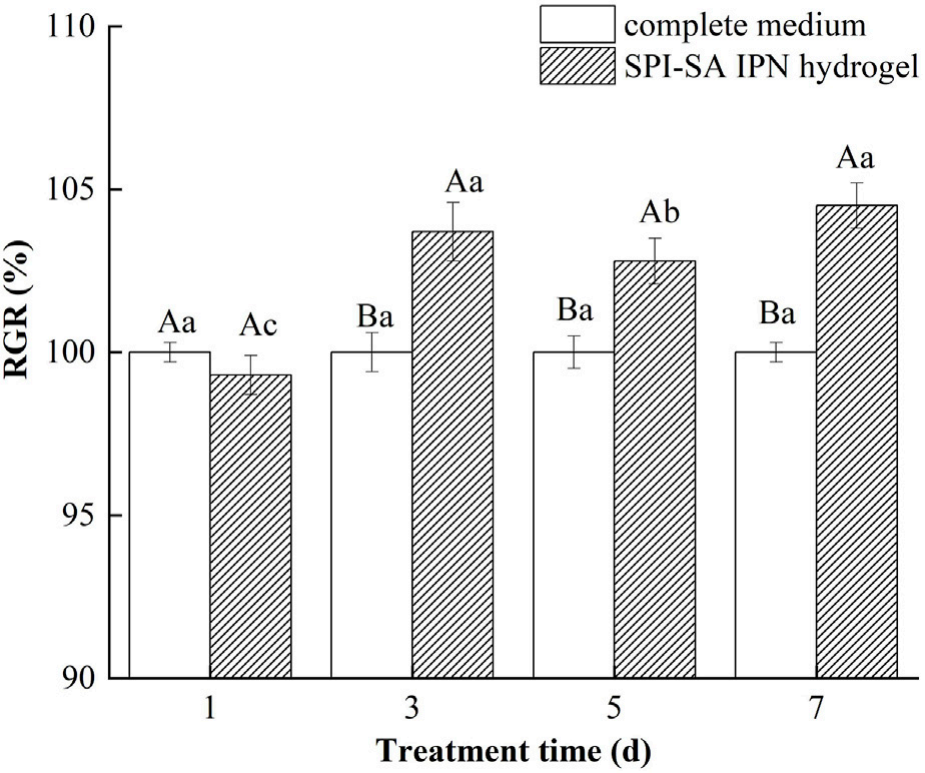
The relative growth rate of cells in SPI-SA IPN hydrogel.

The ideal cell scaffold material should not release toxic products or produce adverse reactions, which can be assessed by *in vitro* cytotoxicity assays (Elsayed et al., 2023). The leaching method uses complete cell culture medium to leach the samples proportionally and the leachate is contacted with the cells, and the relative cell proliferation rate of L929 cells on SPI-SA IPN hydrogel scaffolds for 1, 3, 5, and 7 days was determined by the CCK-8 method according to the criteria in Table 1 to determine whether the SPI-SA IPN hydrogel scaffolds had toxic effects on the cells, and the results were shown in Figure 10.

**TABLE 1:** Comparison table of material toxicity score (ISO 10993.12-2005).

(RGR,%)	Toxicity level
≥100	0
75–99	1
50–74	2
25–49	3
1–44	4

The cell proliferation rate of L929 cells in SPI-SA IPN hydrogel extract after 1 day of culture decreased compared with the control group (complete medium), decreasing to 99.3%, and the cytotoxicity grade was grade 1. This may be due to the cells were newly exposed to the extract and the original culture medium environment was different, so the cell growth and proliferation were inhibited. However, at the third, fifth, and seventh days, The relative proliferation rate of L929 cells in the immersion solution was significantly higher than that of the control complete medium with a relative cell proliferation rate greater than 100% and a cytotoxicity grade of 0. This indicated that the growth rate was higher than that of the SPI control, because of the hydrogel immersion solution contained polysaccharides and proteins that could promote cell growth. The relative cell proliferation rate reached 103.7% ± 0.9%, 102.8% ± 0.7% and 104.5% ± 0.7% at the culture of 3, 5, and 7 days within the hydrogel extract, which was increased significantly (*p* < 0.05) compared with that of the culture at 1 day (99.3% ± 0.6%), indicating that the cells showed better cell growth within the hydrogel extract. However, the value of relative cell proliferation rate of L929 cells cultured within SPI-SA IPN hydrogel extract at day 5 decreased slightly, and the relative cell proliferation rate at day 7 was not significantly different from that at day 1 (*p* > 0.05), which could be due to the fact that the presence of SPI promoted cell growth and division, but due to the limited nutrition of the cell culture environment the cells were too numerous after a certain time. L929 cells began to compete with each other for nutrients and growth space in the culture medium, resulting in contact inhibition, so that the relative cell growth rate was reduced on day 5 and the relative cell growth rate on day 7 was less different from that on day 3.

The relative cell proliferation rate of L929 fibroblasts in the hydrogel extracts indicated that the degradation products of the hydrogels were non-toxic and the SPI-SA IPN hydrogel scaffolds prepared were non-toxic (Zhang et al., 2023). The growth and proliferation of cells within the SPI-SA IPN hydrogel extracts was improved compared to L929 cells grown within the complete medium, the SPI-SA IPN hydrogel scaffold had no negative effect in changing the medium composition, and L929 cells had a higher viability on SPI-SA IPN.

## 4 Conclusion

Taken together, the results of this study showed that the SPI-SA IPN hydrogel prepared by IPN technology has better mechanical properties, more stable structure and less biodegradable than SPI hydrogel, and can culture L929 and C2C12 cells on it. The results of cell growth experiments shown that both cells can grow in IPN hydrogel and have good bio-compatibility, indicating that the IPN hydrogel supports cell growth in the pores of the material. However, the hydrogel with pore size of 100–300 µm prepared in this experiment is more suitable for the growth of L929 cells with cell size of about 20–50 µm compared with C2C12 cells with cell size of about 100–300 µm. The cell proliferation rate of L929 fibroblasts in hydrogel extract showed that the degradation products of hydrogel were nontoxic, suggesting that the SPI-SA IPN hydrogel scaffold was nontoxic. These results shown that SPI-SA IPN hydrogel is a candidate for cell growth *in vitro*.

## Data Availability

The raw data supporting the conclusions of this article will be made available by the authors, without undue reservation.
